# Analysis of the features and source gene composition of the AluYg6 subfamily of human retrotransposons

**DOI:** 10.1186/1471-2148-7-102

**Published:** 2007-07-01

**Authors:** Pamela Styles, John FY Brookfield

**Affiliations:** 1 Institute of Genetics, School of Biology, University of Nottingham, Queens Medical Centre, Nottingham, UK

## Abstract

**Background:**

Alu elements are a family of SINE retrotransposons in primates. They are classified into subfamilies according to specific diagnostic mutations from the general Alu consensus. It is now believed that there may be several retrotranspositionally-competent source genes within an Alu subfamily. To investigate the evolution of young Alu elements it is critical to have access to complete subfamilies, which, following the release of the final human genome assembly, can now be obtained using *in silico *methods.

**Results:**

380 elements belonging to the young AluYg6 subfamily were identified in the human genome, a number significantly exceeding prior expectations. An AluYg6 element was also identified in the chimpanzee genome, indicating that the subfamily is older than previously estimated, and appears to have undergone a period of dormancy before its expansion. The relative contributions of back mutation and gene conversion to variation at the six diagnostic positions are examined, and cases of complete forward gene conversion events are reported. Two small subfamilies derived from AluYg6 have been identified, named AluYg6a2 and AluYg5b3, which contain 40 and 27 members, respectively. These small subfamilies are used to illustrate the ambiguity regarding Alu subfamily definition, and to assess the contribution of secondary source genes to the AluYg6 subfamily.

**Conclusion:**

The number of elements in the AluYg6 subfamily greatly exceeds prior expectations, indicating that the current knowledge of young Alu subfamilies is incomplete, and that prior analyses that have been carried out using these data may have generated inaccurate results. A definition of primary and secondary source genes has been provided, and it has been shown that several source genes have contributed to the proliferation of the AluYg6 subfamily. Access to the sequence data for the complete AluYg6 subfamily will be invaluable in future computational analyses investigating the evolution of young Alu subfamilies.

## Background

Alu elements are a family of SINE retrotransposons found in primates, which have been propagated non-autonomously by utilising the enzymatic machinery of autonomous L1 LINE elements [1,2]. Alu elements are approximately 300 bp in length, and have proliferated by the process of retrotransposition [3] to over one million copies ([4] in the human genome, comprising approximately 11% of the genome by mass [5].

The majority of these elements were generated 35–60 mya during the peak of Alu retrotranspositional activity [5], which has subsequently reduced to the current, relatively low level. Despite their high copy number, only a relatively small number of Alu elements are capable of generating new copies [6]. This has led to the generation of a collection of Alu subfamilies of differing ages, characterised by diagnostic mutations [7]. These correspond to mutations present within the source genes that gave rise to each subfamily.

The term "source gene" is used to describe an Alu element which is both transcriptionally and retrotranspositionally active, and therefore capable of producing daughter elements. It is known that there are several currently active Alu source genes, each of which has given rise to a "young" Alu subfamily. Several of these young subfamilies, including AluYg6 [8], have arisen so recently that subfamily members have only been identified in the genomes of humans, and not of non-human primates.

It has been reported previously that approximately 10–20% of elements within a young Alu subfamily may operate as secondary source genes [9]. It has also been estimated that there may be at least 143 Alu source genes in total, which would require many active elements within each of the currently-defined subfamilies [10].

Following the release of the finalised human genome assembly, it should now be possible to obtain, by *in silico *methods, complete sets of Alu sequences belonging to each subfamily. However, as the young Alu subfamilies are actively retrotransposing, there are likely to be additional polymorphic elements which are not present in the human genome database. Here, we report the identification of 380 Alu elements belonging to the AluYg6 subfamily, and the subsequent detection of two new small Alu subfamilies derived from AluYg6. This represents a substantial improvement in our knowledge of this subfamily, of which 156 members have been previously reported [8]. These data, which represent a complete Alu subfamily, will be extremely useful in future computational studies investigating Alu subfamily evolution, by methods such as those which have recently been reported [9,11]. In light of these data, the issue of defining what constitutes an Alu subfamily is addressed, along with the criteria that should be followed in assigning an element to a particular subfamily.

## Results

### AluYg6 copy number, distribution and sequence features

**Figure 1:**
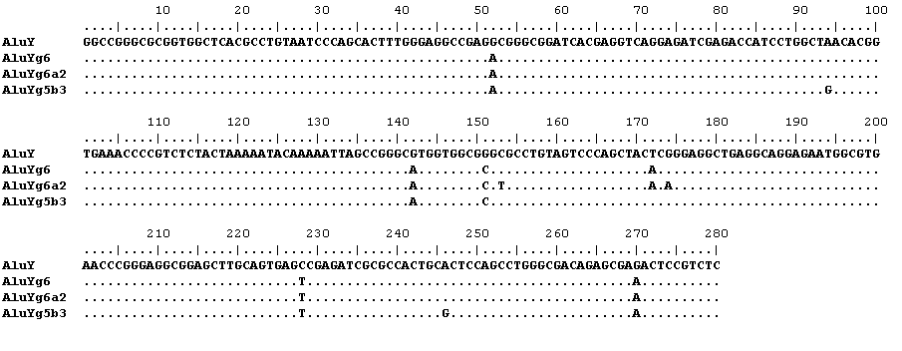
**Alignment of the consensus sequences of the AluY, AluYg6, AluYg6a2 and AluYg5b3 subfamilies**. Diagnostic mutations are shown for the three younger subfamilies. Identical nucleotides are represented by dots.

The AluYg6 subfamily consensus is 281 bp in length, and is characterised by six diagnostic changes from the AluY consensus (see figure 1). A total of 380 AluYg6 elements were extracted from the human genome (see Additional files 1 and 2). 281 of these possessed all six AluYg6 diagnostic mutations, including 23 that matched the AluYg6 consensus perfectly. In additional to these 281, a further 11 elements exhibited 5 of the diagnostic mutations, with a non-ancestral change at the final position, which can be assumed to have been generated by a forward mutation event in each of these 11 elements at the diagnostic position. This generates a set of 292 Alu elements which have unequivocally been derived from a source gene of the AluYg6 subfamily.

The other 88 elements show an ancestral base, in other words that found in the AluY consensus, at one or two of the diagnostic positions. Such elements may have been generated by back mutation, gene conversion, or more likely a mixture of these two processes. Of these 88 sequences, 71 showed an ancestral base at only one of the six diagnostic sites. In 29 cases, this single diagnostic change was the presence of an ancestral T at position 172, however, this large number can be explained by the inference of a new source gene carrying this mutation (see below). Only four of these 29 sequences do not appear to have been derived from this source gene, and are included in table 1.

**Table 1:** Frequency of ancestral bases in AluYg6 elements with one diagnostic change.

Position	Ancestral base	Yg6 base	Nature of back mutation	Occurrence
52	G	A	Transition	8
142	G	A	Transition	10
151	G	C	Transversion	4
172	T	A	Transversion	4
228	C	T	Transition	7
270	G	A	Transition	13

The ancestral base 270G occurs most frequently of the six ancestral bases in these single diagnostic position variants. This might suggest that mutation of the ancestral G to an A at this position was the final mutation to occur along the AluYg lineage, and that some of these sequences represent intermediate "AluYg5" elements. However, if elements with two diagnostic changes are also considered, 142G is the most common ancestral base, occurring 20 times in total, with 270G being the second most common (15 times in total).

The pattern of integration of AluYg6 elements is not random with respect to chromosomal distribution (chi-squared test, p < 0.01). The number of elements observed and expected on each chromosome is shown in table 2. As expected for a young Alu subfamily [12], AluYg6 elements appear to integrate preferentially into AT-rich DNA.

**Table 2:** The distribution of AluYg6 elements. Observed and expected numbers of AluYg6 elements are shown for each chromosome, based on the total number of AluYg6 elements, and the relative size of each chromosome. Significance is assessed by chi-squared tests comparing the number on each chromosome in turn with numbers on other chromosomes.

Chromosome	Observed	Expected	Significant?
1	19	30	Y
2	31	29	
3	29	25	
4	32	23	
5	30	22	
6	25	21	
7	28	19	Y
8	28	19	Y
9	18	17	
10	21	17	
11	18	17	
12	17	16	
13	5	14	Y
14	11	13	
15	9	13	
16	6	11	
17	11	10	
18	11	10	
19	4	8	
20	7	8	
21	5	6	
22	2	6	
X	12	19	
Y	1	7	Y

5' truncations are relatively common in AluYg6 elements, brought about by incomplete reverse transcription or by imprecise integration [8]. They do not represent post-integration deletion events. 35 AluYg6 elements were truncated at the 5' end, with truncations ranging from 5 to 67 bp. The mean length of these truncations is 30 bp, and the modal length, exhibited by 5 elements, is 36 bp.

**Figure 2:**
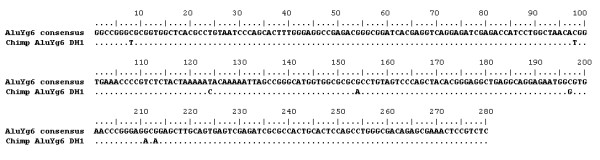
Alignment of the chimpanzee AluYg6 sequence (DH1) and the human AluYg6 consensus.

In contrast to expectations, one example of an AluYg6 element was identified in the chimpanzee. This element possesses all six AluYg6 diagnostic mutations, along with seven additional mutations (see figure 2). Only one of these seven is found in the consensus sequence of another Alu subfamily on RepBase Update [13]. This is the 124T to C mutation, which is found in the AluYf2 consensus. Four of the additional mutations are CpG transitions. We therefore believe that the element is much more likely to have integrated as an AluYg6 than any other subfamily. If it had not integrated as an AluYg6, the six diagnostic mutations would have had to occur by chance, which is unlikely as these are not particularly common mutations, for example, none are CpG transitions. This AluYg6 is not present at the orthologous region in the human genome, where there is only one copy of the target site duplication.

### Frequency of back mutation and partial gene conversion

Alu elements, probably as a consequence of their high copy number, undergo relatively frequent gene conversion events [14]. Gene conversion is a non-reciprocal recombination process, whereby one sequence is converted such that it is identical to a highly similar template sequence, which itself remains unchanged. Gene conversion events involving Alu elements can be complete, whereby the entire element is converted, or partial, such that only a short stretch of sequence within the element is affected.

To look for evidence of partial gene conversion events, the frequency of putative back mutations at the six diagnostic positions (for elements with one or two diagnostic changes) was compared to the frequency of the other possible mutations at these sites. Changes to the ancestral AluY base are greatly overrepresented relative to the alternative two bases at each position, except in the case of 151C, which shows the ancestral G in 8 cases, and a non-ancestral T in 15 cases. However, this site is within a CpG dinucleotide, which explains why a T is seen so frequently at this position. CpG transition mutations occur at approximately six times the rate of non-CpG mutations [15] due to spontaneous deamination of 5-methylcytosine to thymine, resulting in a paucity of CpG, and an excess of TpG and CpA dinucleotides, as in this case. It is also noteworthy that although the transversional change to the ancestral nucleotide is seen 8 times, the alternative transversion is not seen at all.

Perhaps the best evidence supporting the occurrence of partial gene conversion events is at position 172. Excluding elements carrying the ancestral mutation believed to have arisen in another source gene, as above, 8 back mutations are seen, which would represent transversional changes. There were no instances of the other transversion (A to C) seen at this position, and only four instances of the transition mutation.

### Complete gene conversion

**Figure 3:**
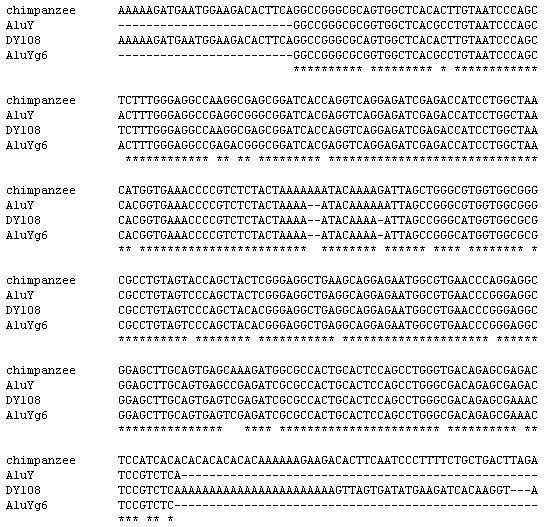
**Alignment of human DY108 (and flanking sequence) with the orthologous region from the chimpanzee**. The consensus sequences for AluY, found in the chimpanzee, and AluYg6 are also shown. For the alignments of the other five previously unreported complete gene conversions see Additional files 3, 4, 5, 6, 7. Alignments were performed with ClustalW using default settings, following by manual editing.

For nine of the elements identified, an Alu was present at the orthologous locus in the chimpanzee, indicative of a complete gene conversion event. This is where an Alu element belonging to an older subfamily has been converted to an AluYg6 along the human lineage. As well as the three complete gene conversion events previously reported [8], six more were identified. In five of these cases (DY108, DY178, DY198, DY285 and DY364), a complete Alu element is present in the chimpanzee (see figure 3).

The final case, DY184, is more ambiguous, as only the left monomer and a short section of the right monomer of an AluSx element are present in the chimpanzee. The human AluYg6 sequence is flanked at the 3' end by a 17 bp region of homology to the part of the right monomer that remains in the chimpanzee. It is likely that homologous recombination has occurred between these two 17 bp regions along the chimpanzee lineage, causing most of the 3' end of the AluSx to be deleted, and leaving only one copy of the homologous region.

In two cases (DY178 and DY184), the gene conversion event appears to be complete. However, in the other four cases, one ancestral base is present in the AluYg6 sequence (52G), suggesting gene conversion tracts of approximately 200 bp have converted the majority of the sequence, but the beginning of the element is still ancestral.

### Identification of new subfamilies derived from AluYg6

#### 1. AluYg6a2

40 elements were identified with all six of the AluYg6 diagnostic mutations along with two additional mutations (153T and 174A). Both of these mutations are CpG transitions, and may therefore be expected to occur frequently in the data without the inference of a new source gene. However, based on the frequency of these mutations occurring independently in the rest of the data, it was found that these two mutations are found together within a single element significantly more often than would be expected due to parallel mutation (chi-squared test, p < 0.01), therefore suggesting a source element containing these two mutations is responsible for their propagation. We will refer to these elements as Yg6a2, according to the standard nomenclature for Alu elements [16]. An unfilled site was seen at the orthologous locus in the chimpanzee genome for all Yg6a2 elements.

The CpG mutation at position 153 occurs 33 times in the rest of the data (a total of 73 times including Yg6a2 elements), whereas the CpG mutation at position 174 only occurs twice in the rest of the data (42 times in total). This might suggest that the 153C to T mutation occurred first, within the source gene, and was propagated before the 174G to A mutation took place within the same source gene.

#### 2. AluYg5b3

27 elements were identified which have five diagnostic mutations of the AluYg6 subfamily, along with two additional mutations. These mutations occur significantly more frequently together than alone in the complete Yg6 dataset, suggesting these mutations are shared by descent from a new source gene, rather than by multiple parallel mutation events. Interestingly, a further mutation is shared by seven of the Yg5b3 elements, which is only seen in 15 of the 353 remaining AluYg6 elements. This may indicate that this mutation has occurred in the Yg5b3 source gene and has been subsequently proliferated. However, the mutation is a C to T transition occurring within a CpG dinucleotide, which may have arisen multiple times independently. An unfilled site was seen at the orthologous locus in the chimpanzee genome for all Yg5b3 elements.

One of the three mutations that are diagnostic for this new subfamily is a back mutation at one of the six AluYg6 diagnostic sites (172A to T). Yg5b3 is therefore an appropriate designation for this subfamily, as it contains 5 of the diagnostic mutations of the AluYg lineage, with 3 mutations which distinguish it from its ancestral sequence, AluYg6. It is also possible that this subfamily may be derived from an intermediate AluYg5, which began retrotransposing following the occurrence of the two additional mutations.

Active Alu source genes generally appear to have retained high numbers of CpG dinucleotides [17], which will have degenerated to TpG and CpA in inactive elements. As CpG dinucleotides are prone to rapid degeneration, elements with no CpG mutations may represent recent transpositions. The AluYg6 subfamily consensus sequence contains 25 CpG dinucleotides, compared to 26 in AluYg5b3. This high level of CpG may be related to the activity of the source gene.

### Additional source genes

Although no other source genes within the AluYg6 subfamily have propagated to the extent of the two described above, there are another two groups of elements for which inference of secondary source genes is a possible explanation for their shared mutations. 23 elements were identified which have a C at position 277, instead of the T found in the Yg6 consensus. This would represent a transitional mutation, and may therefore be expected to occur relatively frequently, but it is highly overrepresented relative to other transition mutations. The second group contains only two elements, but the rarity of the mutations they share makes parallel mutation unlikely. Elements DY380 on chromosome 18 and DY383 on chromosome 19 both show a G to A mutation at position 11, a 3-mer expansion of the middle A-rich tract, and a two nucleotide insertion ("AC") at position 173. Although the other two mutations are relatively common, small insertions into AluYg6 elements, which do not correspond to poly(A) tract expansion, are extremely rare, occurring in only six other elements out of 380. Out of these six cases, only one shows a dinucleotide insertion. Four possess single nucleotide insertions and the final an eight nucleotide duplication.

## Discussion

Here we have reported the identification of 380 members of AluYg6 and its derivative subfamilies. It has been estimated previously [8] that this family would contain 173 elements, a number which has been greatly exceeded by this work.

It appears that a young Alu subfamily can be defined in two ways, either by its present-day sequence, or by its ancestry (that is, its evolutionary history at its genomic locus since the moment of integration). A subfamily may be described as a collection of elements with the specified base at defined diagnostic positions. In this case, any Alu element with those diagnostic bases would be defined as a member of that subfamily. Such a definition would result in a set of 281 AluYg6 elements from these data.

Alternatively, an Alu element can be defined as belonging to a subfamily if it is reasonable to assume that at the moment of integration, the sequence corresponded to that of the subfamily source gene, which may have since undergone gene conversion or back mutation such that it might show diagnostic changes. For example, it is feasible that many, if not all, of the elements presented here with only one diagnostic change, integrated into the genome as elements with all six AluYg6 diagnostic bases. Their inclusion in the subfamily acknowledges aspects of the evolutionary history of the AluYg6 subfamily that would otherwise be ignored. Therefore, for evolutionary analyses, the inclusion of an element within a subfamily based on its inferred state at the moment of integration seems more appropriate than inclusion based solely on its present-day sequence.

In the cases of elements showing an ancestral base at one of the diagnostic positions, it was found that 270G was the most frequent of these bases, which might suggest that this was the last position to change along the AluYg lineage. If the ancestral "AluYg5" source gene contained a G at this position, it is possible that some of these elements represent intermediates in the formation of AluYg6 rather than AluYg6 elements that have mutated, which might explain the relatively high frequency of this mutation. However, there is not much evidence in favour of this, as when elements with either one or two ancestral diagnostic positions are considered, 142G is the most common ancestral nucleotide. Both 52G and 142G occur frequently in single position variants, and the lower frequency of both 151T and 172T can be explained by the relative unlikelihood of back mutations at these positions, as these would require transversional rather than transitional changes. The fact that there is not much difference in the frequency of the ancestral bases among AluYg6 elements with one diagnostic change might indicate an absence of intermediates, suggesting that the AluYg lineage did not become retrotranspositionally active until all six diagnostic changes had occurred.

It was found that ancestral bases were overrepresented at diagnostic positions in elements with one or two diagnostic changes relative to other non-Yg6 bases. This suggests at least some instances of partial gene conversion, whereby short gene conversion tracts have modified part of an AluYg6 insertion using an older Alu element as a template. It was also found that the ancestral bases that could be generated from the AluYg6 diagnostic bases by transition mutations were more common than those that could be generated by transversions. Taken together, these two observations suggest that both processes, back mutation and partial gene conversion, have each in some way contributed to the diversity seen among elements of the AluYg6 subfamily.

In nine cases where an AluYg6 was present in the human genome, an older Alu element was present at the orthologous locus in the chimpanzee genome. In the six previously unreported cases, four exhibit a short stretch of bases at the beginning of the element which correspond to the older Alu element, including the ancestral base G at position 52. These four cases can be inferred to represent almost complete gene conversion events, although in the absence of the element in the chimpanzee, these would more likely be interpreted as short gene conversion tracts having converted a stretch of bases at the beginning an AluYg6 using an older Alu as a template. This alternative explanation is still possible, as the element in the chimpanzee may represent a parallel insertion, and the site may have been unfilled in the human-chimpanzee ancestor. An AluYg6 would then have inserted along the human lineage, which was then partially converted to AluY. If this were the case, given that all four confirmed partial gene conversion tracts cover the 5' end of the sequence, it might suggest a preference for gene conversion tracts forming in this region. A preference for gene conversion of the beginning of the element may be explained by a greater degree of homology among Alu elements in this region (see Methods). Information regarding the nature of this site in the genomes of other African apes would help to resolve this issue.

In the other two cases, an older Alu in the chimpanzee appears to have been replaced entirely with an AluYg6 along the human lineage. Again in these two cases, it is possible that parallel insertion, rather than gene conversion, is responsible for this observation. This is, however, unlikely in the case of element DY184, as the element in chimpanzee belongs to the AluSx subfamily, which is believed to only be currently retrotransposing at extremely low levels [18].

It is likely that there are examples of complete gene conversion that cannot be detected. For example, such events may have generated AluYg6 elements from other young Alu elements, which would not be present in the chimpanzee. It is also possible that backward gene conversion events have occurred, whereby, following its insertion, an AluYg6 has been converted to an older Alu element, and therefore cannot be identified as an AluYg6 insertion.

One AluYg6 element was identified in the chimpanzee, which was absent from the orthologous region in the human genome. This element may have transposed to its current location in the chimpanzee following the human-chimpanzee divergence, which would indicate that there is at least one other AluYg6 in the chimpanzee. Alternatively, it is possible that this Alu was present in the human-chimpanzee ancestor and has been precisely deleted by recombination between the flanking direct repeats along the human lineage, a property of Alu elements that has been identified before [19]. Alternatively, this AluYg6 may have been polymorphic in the ancestral population, and has been fixed in the chimpanzee but lost by drift in humans. Regardless of which of these explanations is correct, this finding shows that the first AluYg6 element must have arisen further into the past than previously estimated [8], although the subfamily may have undergone a period of relative dormancy with respect to its retrotranspositional rate [20], only proliferating to considerable numbers along the human lineage following the human-chimpanzee divergence. It has previously been suggested that the evolution of a successful subfamily progenitor sequence occurs well in advance of its peak activity [21]. These authors also note the lower levels of young Alu insertions in the chimpanzee relative to the human genome, and suggest a general increase in retrotranspositional activity in humans as the most favourable explanation.

Two groups of elements were identified that appear to be derived from source genes which do not correspond to the AluYg6 consensus sequence. This confirms the existence of "secondary" source genes within what has previously been considered a single subfamily. However, the idea of secondary source genes is poorly defined, as unless such source genes were identical to the original subfamily consensus, they would propagate diagnostic mutations themselves. This would generate small collections of elements which can themselves be considered new subfamilies, as we have shown here. It is not clear at what point a source gene with a mutation from the consensus of the subfamily from which it has arisen should cease to be regarded as a secondary source gene of its ancestral subfamily, and be considered a primary source gene and consensus sequence for a new derivative subfamily. We propose that where a source gene can be seen to be producing daughter elements with unique mutations relative to the ancestral source gene (in this case, AluYg6), these daughter elements should be considered a derivative subfamily. Such subfamilies would still be considered as members of the ancestral subfamily for the purposes of evolutionary analyses. However, inclusion of these sequences in studies where the mutational variation from the subfamily consensus seen among the elements is used to make inferences about their evolution (for example, in estimating the age of a subfamily), would artificially inflate the total number of mutations seen, as some of these changes have been propagated by retrotransposition rather than mutation.

It is likely that there are further secondary source genes operating in the AluYg6 subfamily. This is suggested by the high frequency of the 277C mutation, and the sharing of a rare mutation by DY380 and DY383. In the latter case, it is quite likely that gene conversion is responsible for the shared variation in these two elements rather than retrotransposition. This may be a more favourable explanation as the mutations are shared by only two elements, although these sequences could suggest a source gene active at very low levels. The high frequency of the 277C mutation is much more likely to represent the activity of another source gene, although alternative explanations are also possible, such as a high rate of mutation at this site. However, if this were the case, the other non-ancestral nucleotides (A and G) would also be expected to be seen at high frequency, and this is not the case. It is possible that this mutation has occurred frequently by chance, and with only one mutation shared between elements it is harder to distinguish between source gene activity and parallel mutation.

It would be interesting to determine the level of polymorphism for each of the AluYg6 elements identified, particularly those originating from the two "new" source genes. Polymorphism data might provide information regarding the relative ages of the elements, which would help to determine whether the AluYg5b3 and Yg6a2 subfamilies have arisen recently, or whether they were derived from AluYg6 relatively shortly after the Yg6 subfamily itself began to retrotranspose, and have simply been less effective at propagating themselves, hence their low copy number. AluYg6a2 elements generally have a high level of identity to their consensus sequence, with approximately 33% (13/40) showing perfect identity to the consensus. This might suggest a relatively recent origin for this subfamily. In contrast, only around 7% (2/27) of AluYg5b3 elements are identical to their subfamily consensus, which is a similar proportion to the AluYg6 subfamily in general. The fact that there are proportionately fewer elements in the Yg5b3 subfamily that are identical to their consensus might suggest that they have been around for some time and simply retrotranspose relatively inefficiently. This information would contribute to our understanding of how the diagnostic mutations within an Alu source gene affect its retrotranspositional success. Some polymorphism data is already available for the AluYg6 subfamily, and suggests polymorphism levels of around 10% [8].

It is unlikely that either of these two subfamilies represent mutations occurring in the AluYg6 source gene, as recent retrotranspositions of this gene have been identified in the form of low frequency polymorphic insertions [8].

## Conclusion

The AluYg6 subfamily began its expansion along the human lineage between three and four million years ago [8] following the divergence of humans and chimpanzees, but the initial Yg6 sequence appears to have originated prior to this event. The source genes for this subfamily have generated a copy number of at least 380 elements, some of which have mutated or undergone partial gene conversion events to generate the level of diversity seen today. We have shown that there at least three active source genes within the AluYg6 subfamily, two of which have given rise to the new small subfamilies AluYg6a2 and AluYg5b3. It is possible that there are other active elements within this subfamily, which are harder to detect. This would be the case if these source genes were identical to the AluYg6 consensus, had arisen recently, or were relatively inefficient at retrotransposition. We have used these two small subfamilies to illustrate the ambiguity regarding Alu subfamily definition, and have proposed a more rigorous definition. We believe that having access to the sequence data for a complete young Alu subfamily will be useful for exploring new computational methods for investigating the evolution of young Alu elements, in particular, developing new methods of modelling the subfamily amplification process, and that further work will improve our understanding of the evolution of Alu subfamilies and the impact of secondary source genes.

## Methods

A BLASTN [22] search was conducted using a query sequence corresponding to bases 52–271 of the AluYg6 consensus sequence, which was obtained using RepBase Update [13]. This query sequence was chosen as it contains all six diagnostic mutations for the Yg6 subfamily, while excluding superfluous sequence from the 5' and 3' ends of the consensus which would have increased the number of hits corresponding to Alu elements belonging to other subfamilies. For example, the first 47 bp of the Alu consensus sequence are identical in the consensus sequences of all but three of the very youngest Alu subfamilies – Yd3, Yd3a1 and Yi6, which contain a C to T transition at position 23 [13]. Use of this query sequence also reduced the chance of missing a genuine AluYg6 with a substantial 5' truncation. Default BLASTN search parameters were used with the exception of word size (W), which was increased to 15, again to attempt to reduce the number of hits not corresponding to AluYg6 elements.

Each result was examined to check for the presence of the Yg6 subfamily diagnostic mutations. Results were discarded which did not possess the correct base at these diagnostic positions. Results which possessed four or five of the diagnostic mutations were retained to investigate the possibility of partial gene conversion events.

For each result that was retained the sequence corresponding to the BLASTN hit, i.e. positions 52–271 of the Yg6 element, was extracted, along with 1000 bp of both 5' and 3' flanking DNA sequence. Flanking sequences were extracted to enable identification of orthologous regions in the chimpanzee genome. This yielded a collection of 2220 bp DNA sequences, each containing an AluYg6 element. These were then screened for duplicates, which were subsequently discarded.

Each 2220 bp extracted sequence was used as a query sequence in a BLAST-like Alignment Tool (BLAT) [23] analysis of the chimpanzee genome assembly [24]. This was done to identify the orthologous region in the chimpanzee genome, to check for both complete and partial gene conversion events. In the majority of cases, a gap was present in the chimpanzee sequence when aligned with the human sequence, corresponding to the entire AluYg6 element and the target site duplication. Alignments were performed using ClustalW [25] with default parameters. In some cases, an Alu element was found at the same location in the chimpanzee. In all such cases, the Alu element was a member of an older Alu subfamily, which can be inferred to have undergone a gene conversion event along the human lineage, and does not represent a true AluYg6 insertion.

The AluYg6 element within each sequence was aligned to the AluYg6 consensus, then extracted, without its oligo-dA tail. A custom-made Perl program was used to identify any mutations that had occurred in each element relative to the AluYg6 consensus.

## Abbreviations

Mya – million years ago

SINE – Short Interspersed Element

LINE – Long Interspersed Element

## Authors' contributions

This work was carried out by PS as part of her PhD study supported by the BBSRC and supervised by JFYB.

## Supplementary Material

Additional file 1 AluYg6 elements – locations and features. A table of all AluYg6 elements, including their chromosomal location, identity to the AluYg6 consensus, number of CpG dinucleotides and length of any 5' truncation. Click here for file

Additional file 2 AluYg6 sequence alignment. Alignment file of the sequences of all AluYg6 elements. Click here for file

Additional file 3 DY178 alignment. Alignment of the human AluYg6 element DY178 with the orthologous region from the chimpanzee genome, showing complete gene conversion. Click here for file

Additional file 4 DY184 alignment. Alignment of the human AluYg6 element DY184 with the orthologous region from the chimpanzee genome, showing complete gene conversion. Click here for file

Additional file 5 DY198 alignment. Alignment of the human AluYg6 element DY198 with the orthologous region from the chimpanzee genome, showing complete gene conversion. Click here for file

Additional file 6 DY285 alignment. Alignment of the human AluYg6 element DY285 with the orthologous region from the chimpanzee genome, showing complete gene conversion. Click here for file

Additional file 7 DY364 alignment. Alignment of the human AluYg6 element DY364 with the orthologous region from the chimpanzee genome, showing complete gene conversion. Click here for file
