# Supplementary figures and images for: Analysis of the features and source gene composition of the AluYg6 subfamily of human retrotransposons
Source: BMC Evol Biol. 2007 Jul 1;7:102. doi: 10.1186/1471-2148-7-102 (PMC1925064; doi:10.1186/1471-2148-7-102)

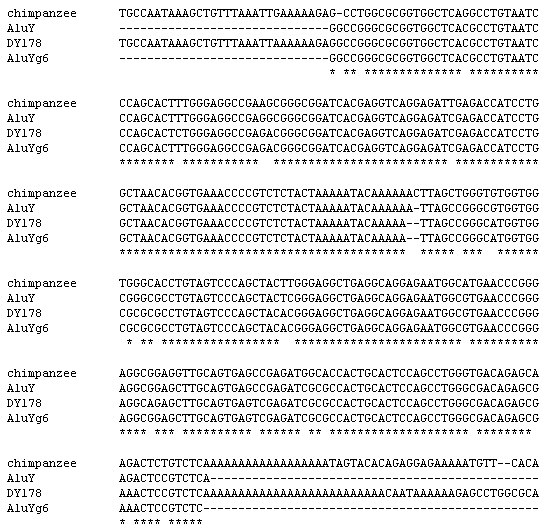

Supplement: Additional file 3 — DY178 alignment. Alignment of the human AluYg6 element DY178 with the orthologous region from the chimpanzee genome, showing complete gene conversion. [file 1471-2148-7-102-S3.tiff]

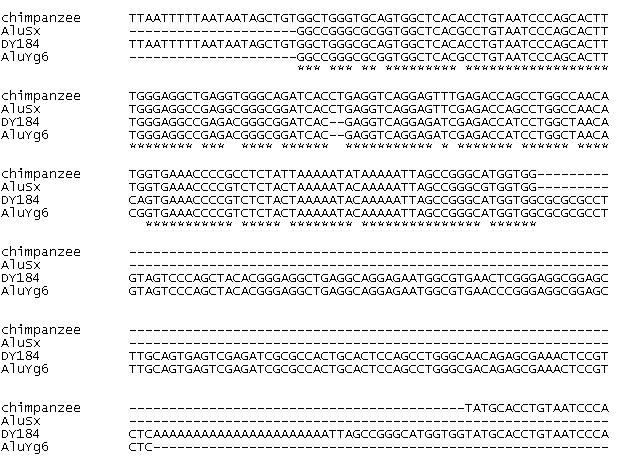

Supplement: Additional file 4 — DY184 alignment. Alignment of the human AluYg6 element DY184 with the orthologous region from the chimpanzee genome, showing complete gene conversion. [file 1471-2148-7-102-S4.tiff]

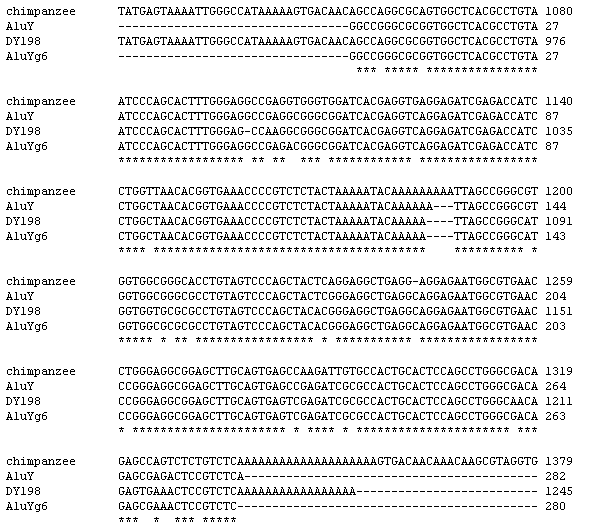

Supplement: Additional file 5 — DY198 alignment. Alignment of the human AluYg6 element DY198 with the orthologous region from the chimpanzee genome, showing complete gene conversion. [file 1471-2148-7-102-S5.tiff]

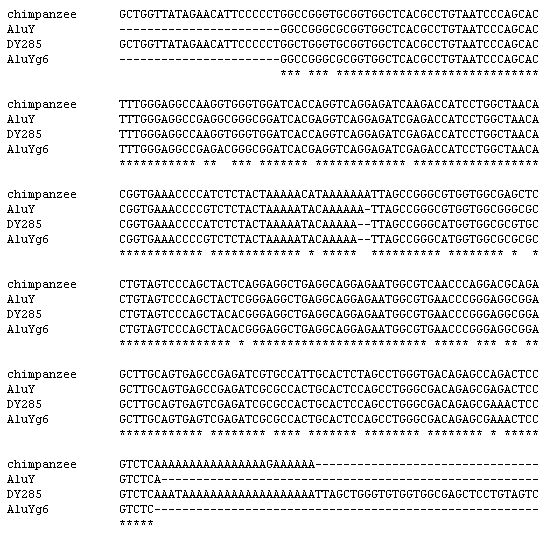

Supplement: Additional file 6 — DY285 alignment. Alignment of the human AluYg6 element DY285 with the orthologous region from the chimpanzee genome, showing complete gene conversion. [file 1471-2148-7-102-S6.tiff]

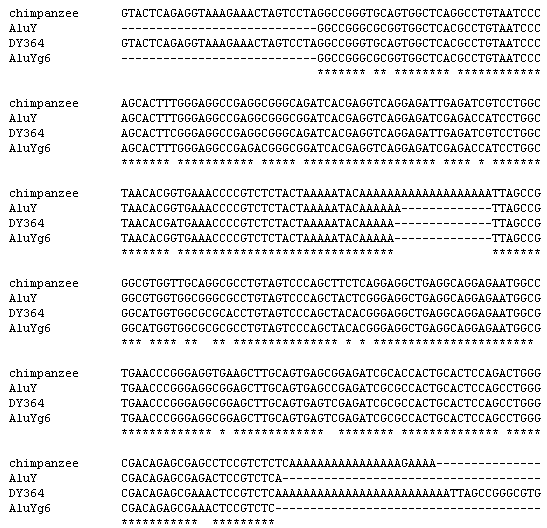

Supplement: Additional file 7 — DY364 alignment. Alignment of the human AluYg6 element DY364 with the orthologous region from the chimpanzee genome, showing complete gene conversion. [file 1471-2148-7-102-S7.tiff]
